# CASPR2 antibody-related neurological syndromes in children: three cases report and literature review

**DOI:** 10.1186/s12887-026-06549-4

**Published:** 2026-02-05

**Authors:** Long-Ying Peng, Shu-Jing Pan, Ren-Ke Li, Juan Li, Jing Chen, Xiao-Hua Yu, Chang-Jian Yang, Xiao-Mei Shu, Mao-Qiang Tian

**Affiliations:** 1 Department of Pediatrics, Affiliated Hospital of Zunyi Medical University, Zunyi, China; 2 Department of Pediatrics, Guizhou Children’s Hospital, Zunyi, China

**Keywords:** Autoimmune encephalitis, Morvan syndrome, Peripheral nerve hyperexcitability, CASPR2, Children

## Abstract

**Background:**

Contactin-associated protein-like 2 (CASPR2) antibody-related neurological syndrome is well defined in adults, while data in children is rare. We perform this case series and literature review to ascertain the clinical features of this disorder in children.

**Case presentations:**

We describe three cases who were diagnosed with CASPR2 antibody-related neurological syndrome, and then systematically characterized clinical features, therapeutic responses, and outcomes in 64 patients in literature. Among the 67 patients, the median age was 8.4 years (range 0.5–18), with a male predominance (65.7%). The most characteristic symptoms of CASPR2 antibody-related neurological syndrome include psychiatric abnormalities (68.7%), sleep disorders (55.2%), dysautonomia (44.8%), movement disorders (43.3%), seizures (41.8%), neuropathic pain (34.3%), and cognitive disturbances (31.3%). Autoimmune encephalitis (AE, 62.7%) was most frequent, followed by Morvan syndrome (MoS, 26.9%) and peripheral nerve hyperexcitability (PNH, 7.5%). CASPR2 antibodies are typically detected in serum (85.1%), but rarely in CSF exclusively (3.0%). Investigations revealed abnormalities in CSF analysis (22.9%; 13 AE, 5 MoS), brain MRI (24.1%; 11 AE, 3 MoS), and EEG (67.3%; 26 AE, 7 MoS). Neoplasm was not found in our case series and literature review. Regarding treatment, 46 patients received first-line immunotherapy alone, while 5 required second-line treatment. Immunotherapy demonstrated highly effective, with 95.8% achieving a favorable outcome (mRS score ≤ 2) and a low recurrence rate of 5.1%.

**Conclusions:**

We therefore recommend that all children presenting with new-onset psychiatric abnormalities, sleep disorders, dysautonomia, movement disorders, seizures, neuropathic pain, and cognitive disturbances should be suspected of having CASPR2 antibody-related neurological syndrome. Neoplastic processes are rare in pediatric patients. Early recognition of the disease is important because it responds well to immunotherapy.

**Supplementary Information:**

The online version contains supplementary material available at 10.1186/s12887-026-06549-4.

## Background

CASPR2, a cellular adhesion protein of the neurexin family, is strongly expressed at the juxtaparanodes of myelinated axons in the central and peripheral nervous systems [1]. It regulates the formation and maintains the stability of distinct axonal domains around the nodes of Ranvier, serving as a membrane scaffold that clusters Kv1 channels at the juxta paranodal region [2]. CASPR2 antibody-related neurological syndromes are classically associated with autoimmune encephalitis (AE), Morvan syndrome (MoS), and peripheral nerve hyperexcitability (PNH) [3]. These syndromes are commonly observed in elderly males (median age 60), with well-documented clinical presentations [4]. However, they are less frequently encountered in children, leading to a lack of well-defined phenotype and understanding of immunotherapy responsiveness in pediatric cases [5, 6]. This field is of major clinical importance to all pediatric neurologists because these patients present with a wide variety of neurological syndromes that are responsive to immunotherapy. So the analysis of the clinical data, to contribute to the definition of clinical features of CASPR2 antibody-related neurological syndrome in children and favor early diagnosis and immunotherapy remains important.

Herein, inspired by three index patients with CASPR2 antibody-related neurological syndrome, we systematically characterized clinical features, therapeutic responses, and outcomes in 67 patients with CASPR2 antibodies, aiming to provide clues for pediatricians to understand the disease. Although data were collected retrospectively, this is the first literature review of pediatric patients summarized with this rare disease so far.

## Case presentations

Patients with clinically suspected neurological autoimmunity were recruited from the Department of Pediatrics, Affiliated Hospital of Zunyi Medical University between January 2015 and December 2023. Serum and CSF samples from each patient were tested using a live cell-based assay (BCA) for CASPR2 antibodies. Over 8 years, 72 pediatric patients were tested in our hospital. A total of 3 anti-CASPR2-positive patients were identified among those examined. Here, we show the cases separately.

### Patient 1

A 5-year-old previously well girl was admitted for abdominal pain and tremulousness on lower limbs of ten days duration. She developed pain, hyperhidrosis, and numbness when her legs cramped, severely restricting her activities. Upon admission, she subsequently presented with a sleep disorder, cognitive decline, behavioral changes, memory impairment, and a lack of interest in her surroundings. There was no history of recent illness or drug ingestion. The child had an uneventful birth history and family history. Upon physical examination, the patient presented with a depressive mood and reduced verbal output. Nonetheless, there were no apparent neurological deficits observed. Electroencephalogram (EEG) showed slow background activity and focal slowing. Magnetic resonance imaging (MRI) of the brain and nerve conduction study were normal. A computed tomography (CT) chest abdomen/pelvis was performed to rule out underlying neoplasms. Cerebrospinal fluid (CSF) analysis revealed no pleocytosis, normal protein and sugar. CASPR2 antibodies were found in the girl’s serum (1:10) on CBA but not in her CSF. She was diagnosed with MoS, and treated with intravenous methylprednisolone (IVMP) for 5 days with intravenous immunoglobulin (IVIG) 2 g/kg. She showed a quick improvement in her condition and returned to her baseline within 2 weeks. Then she was treated with oral prednisolone 2 mg/kg for 6 months and was gradually tapered and stopped. The child has been on follow-up for the last one year and still remains normal.

### Patient 2

A previously well 12-year-old boy presented to our department with a 1-day history of headache and cognitive change, followed by twitching of limb muscles, reduced consciousness, urinary incontinence, sleep disturbance, and meaningless talk. He had no significant past medical, treatment, or family history. On physical examination at admission, he was encephalopathic, with a Glasgow Coma Scale (GCS) of E3V2M4, irritability, and diminished deep tendon reflexes. The EEG showed a generalized slowing in the posterior regions of the head. All investigations including metabolic screening, toxicology screening, thyroid profile, brain MRI, and septic screening were all negative, except for liver aminotransferase elevations (ALT 115U/L, AST 55U/L). Given the patient’s manifestations were suggestive of a paraneoplastic syndrome, we performed tumor markers and imaging studies to rule out an underlying neoplasm, particularly thymoma. CSF cell count and protein concentration were normal. CASPR2 antibody was positive (1:10) in serum but absent in CSF. He was considered to have MoS. His symptoms resolved spontaneously with only symptomatic support (paracetamol and glutathione), beginning to improve three days after onset and returning to normal by day 9. Because of the good symptomatic response, immunomodulation was deferred. He was still free of symptoms on a follow-up visit after 8 years.

### Patient 3

A 5-year-old previously well girl presented with a 2-day history of weakness of the left-sided limb, headache, changes of personality, and abnormal movements in the mouth. There was no numbness or tingling. The child was not showing interest in her surroundings. She had decreased muscle strength (Medical Research Council scale grade 4/5 and 5/5 in left and right limbs, respectively), ataxic gait, and sleep disturbance. Microbiological studies were negative, including fungal and bacterial cultures and polymerase chain reaction for herpes simplex virus, Epstein-Barr virus, and cytomegalovirus. EEG revealed diffuse background slowing. Brain and spine MRI were normal, as was nerve conduction study (NCS). Thyroid function and thyroid peroxidase antibodies were normal. She had undergone an exhaustive workup for neoplasms, which had turned negative. CSF was acellular with normal protein. CASPR2 antibody was detected (1:10) in serum as compared with CSF. Based on the clinical and laboratory findings, she was diagnosed with CASPR2-associated AE and was started on IVMP (20 mg/kg/d for 3 days) and IVIG (2 g/kg in 5 days). After administration of IVMP for 3 days, the child was treated with oral prednisolone 2 mg/kg/d for 6 months. Within 10 days of completing treatment, the patient had completely recovered. She has remained symptom-free for a 16-month follow-up.

In all three patients, comprehensive testing for other neuronal antibodies—including those targeting NMDAR, AMPA1, AMPA2, LGI1, and GABAB receptors—yielded negative results in both serum and CSF.

## Cases in literature

**Fig. 1:**
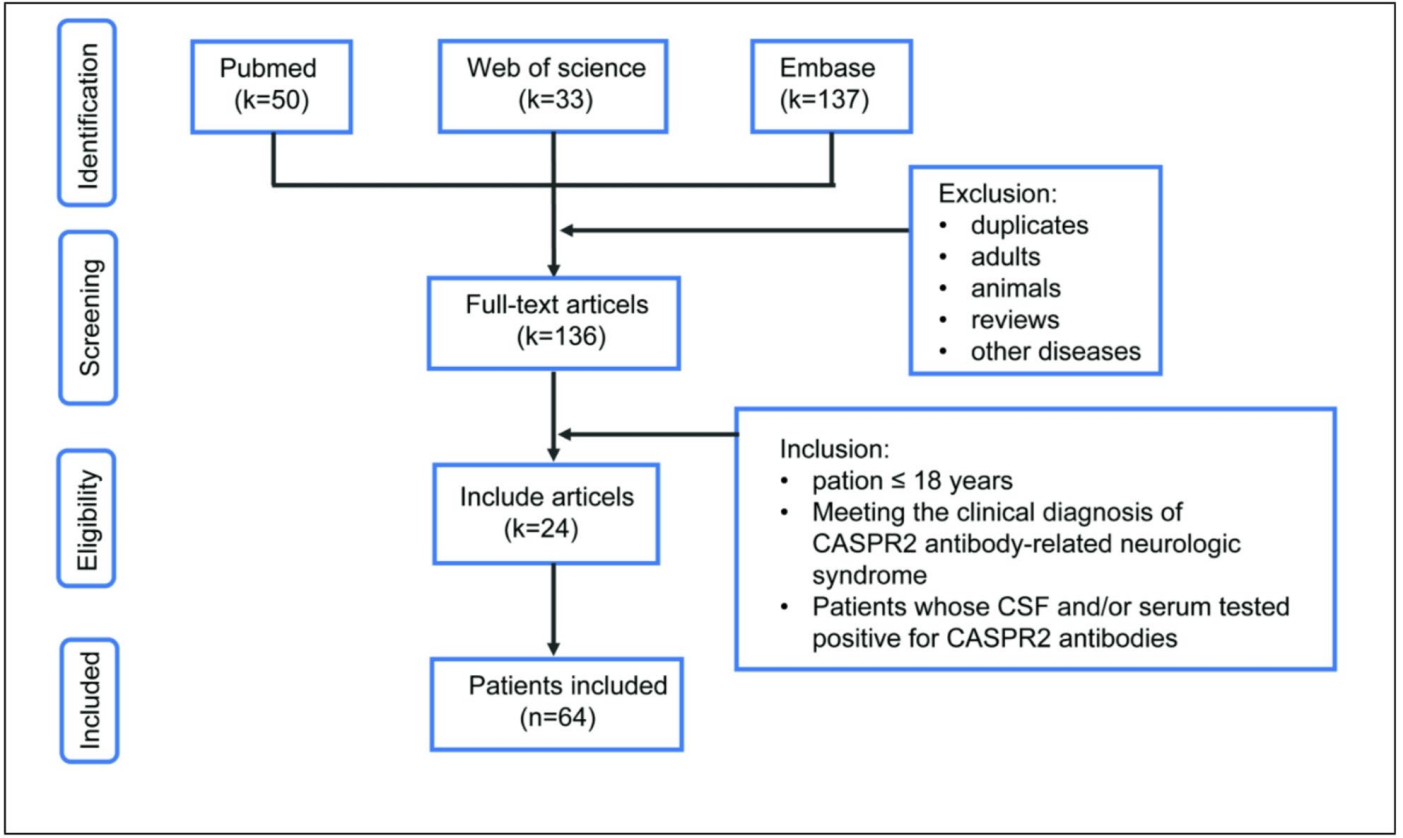
Flow diagram of literature search and study selection process (k = numbers of articles; n = numbers of patients)

The literature search was conducted on Pubmed, Embase, and Web of Science, covering the period from the earliest available records to December 31, 2023. The search utilized the following terms: “CASPR2 or contactin-associated protein-like 2”, “limbic encephalitis”, ”VGKC”, “LGI1”, “children”, “autoimmune encephalitis”, “Morvan syndrome”, and “peripheral nerve hyperexcitability”. Retrieved articles underwent a thorough manual examination to specifically identify pediatric patients (≤ 18 years) with CASPR2 antibodies in CSF and/or serum. Individual patient data was meticulously extracted, with a particular focus on pertinent clinical details (Fig. 1). The primary search identified 220 articles. After removing duplicates and applying the exclusion criteria, 64 pediatric patients with positive antibodies of CASPR2 from the targeted literature were selected for the case series and literature review. Results of the 67 pediatric patients (including 3 cases from our hospital and 64 cases from literature) with CASPR2 antibody-related neurologic syndrome are presented in Table 1.

**Table 1:** Results of the literature review of CASPR2 antibody-related neurological syndrome in children

Characteristics	Values
Demographics	
Males, *n* (%)	44 (65.7)
Median age at onset, y (range)	8.4 (0.5–18)
Categories of neurologic symptoms, *n* (%)	
Morvan syndrome	20 (29.9)
Autoimmune encephalitis	42 (62.7)
Peripheral nerve hyperexcitability	5 (7.4)
Presenting symptoms, *n* (%)	
Psychiatric abnormalities	46 (68.7)
Sleep disorders	37 (55.2)
Dysautonomia	30 (44.8)
Movement disorders	29 (43.3)
Seizures	28 (41.8)
Neuropathic pain	23 (34.3)
Cognitive disturbances	21 (31.3)
Muscle cramping or weakness^a^	15 (22.4)
Weight loss	14 (20.9)
Headache	10 (14.9)
Reduced consiciousness	8 (11.9)
Memory impairment	7 (10.4)
Fever	6 (9.0)
Anti-CASPR2 positivity, *n* (%)^b^	
Serum (with or without CSF)	57 (85.1)
CSF only	2 (3.0)
Both serum and CSF	12 (17.9)
Abnormal MRI, *n* (%)	14 (24.1)
Abnormal CSF, *n* (%)	18 (37.5)
Cell count > 5 cells/µL	11 (22.9)
Protein > 45 mg/dL	10 (20.8)
Abnormal EEG, *n* (%)	33 (67.3)
Slowing	31 (63.3)
Epileptiform discharge	7 (14.3)
Immunosuppressive treatment, *n* (%)	51 (86.4)
First-line immunotherapy	46 (78.0)
Corticosteroids	38 (64.4)
IVIG	38 (64.4)
Corticosteroids + IVIG	32 (54.2)
Plasma exchange	2 (3.4)
Second-line immunotherapy	5 (8.5)
Length of follow-up^c^, m (range)	Median 13
mRS 0, *n* (%)	36 (57.1)
mRS ≤ 2, *n* (%)	46 (95.8)
mRS = 3, *n* (%)	2 (4.2)
Relapse, *n* (%)	3 (5.0)

^a^Among the 67 cases, neuromyotonia was observed in 10 patients (14.9%)

^b^All patients (100%) were positive for anti-CASPR2 antibody

^c^Only 48 out of 67 cases with available modified Rankin Scale (mRS) scores at follow-up

### Patient definition and classification

According to the diagnostic criteria proposed by Graus et al., a diagnosis of AE depends on satisfying all three of the following conditions [7]. First, the patient must present with a subacute onset (rapid progression within 3 months) of symptoms such as working memory deficits, altered mental status, or psychiatric manifestations. Second, there must be at least one objective sign of central nervous system (CNS) involvement, which may include new focal neurological deficits, seizures not attributable to a known pre-existing disorder, CSF pleocytosis (> 5 white blood cells per mm³), or suggestive MRI findings. Finally, other possible causes of the clinical presentation must have been adequately ruled out. MoS was characterized as a combination of cognitive impairment, peripheral nerve hyperexcitability, and dysautonomia or insomnia [8]. PNH was defined as spontaneous muscle overactivity identified either at the physical examination or with electrophysiologic studies [9]. Due to considerable clinical overlap, the diagnostic labels were driven by the predominant symptom complex.

### Demographics and clinical syndromes

**Fig. 2:**
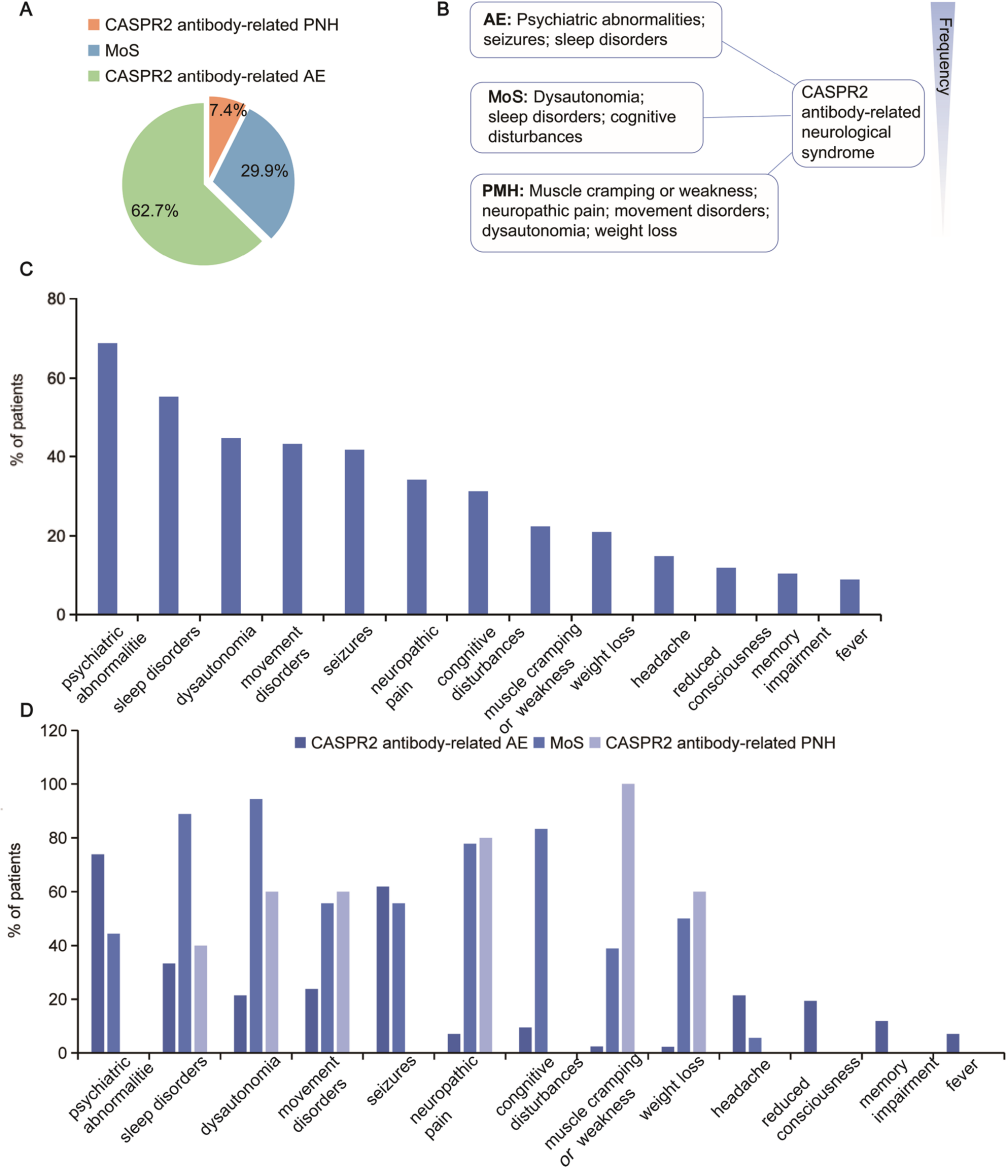
Clinical characterization of CASPR2 antibody-related neurological syndrome in pediatric patients. **A** CASPR2 antibody-related neurological syndrome classification in children. **B** Classic syndromes and the most common characteristic features of CASPR2 antibody-related neurological syndrome in pediatric patients. Listed in an estimated order of descending frequency. **C** Clinical symptoms in patients with CASPR2 antibody-related neurological syndrome. **D** Clinical symptoms in patients with CASPR2 antibody-related AE, MoS and CASPR2 antibody-related PNH. Abbreviation: AE, autoimmune encephalitis; CASPR2, contactin-associated protein-like 2; MoS, Morvan syndrome; PNH, peripheral nerve hyperexcitability

Our case series and literature review included sixty-seven patients (aged 0.5–18 years, median 8 years), among whom 44 (65.7%) were male and 23 (34.3%) were female. CASPR2 antibody-related neurological syndrome is characterized by psychiatric abnormalities (68.7%), sleep disorders (55.2%), dysautonomia (44.8%), movement disorders (43.3%), seizures (41.8%), neuropathic pain (34.3%), cognitive disturbances (31.3%), muscle cramping or weakness (22.4%, including 10 cases of neuromyotonia), weight loss (20.9%), headache (14.9%), reduced consciousness (11.9%), memory impairment (10.4%), and fever (9.0%). The most common presentation in our case series and literature review was psychiatric abnormalities, followed by sleep disorders, dysautonomia, and movement disorders. Less frequent leading clinical symptoms were reduced consciousness, memory impairment, and fever. Forty-two patients met the criteria for CASPR2 antibody-related AE, presenting with psychiatric abnormalities, seizures, and sleep disorders as the most characteristic symptoms. Twenty patients had MoS, which is characterized by dysautonomia (94.4%), sleep disorders (88.9%), cognitive disturbances (83.3%), neuropathic pain (77.8%), movement disorders (55.6%), seizures (55.6%), weight loss (50.0%), psychiatric abnormalities (44.4%), and muscle cramping or weakness (38.9%). Muscle cramping or weakness, neuropathic pain, dysautonomia, movement disorders, and weight loss were the most frequently presenting symptoms in the 5 patients with CASPR2 antibody-related PNH. Symptoms at the last visit included asthenia, seizures, memory and movement impairment, autism spectrum disorder, and mild behavioral change (*n* = 1 each) (Fig. 2).

## Discussion and conclusions

To our knowledge, this is the first literature review of pediatric patients with CASPR2 antibody-related neurological syndrome and it has provided further details about this rare disease. Our review of CASPR2 antibody-related neurological syndrome in children revealed a total of 67 patients, with the age of onset ranging from 0.5 to 18 years, and a median age of 8.4 years. Most patients in our case series and literature review were males (65.7%), which is lower than that reported in adult cases (70–90%), but still in keeping with the previously reported male predominance [10]. Although the incidence is higher in males, our case series and literature review found that a considerable proportion (34.3%) of pediatric patients were female. To avoid missed diagnoses due to gender bias, clinicians should consider this diagnosis in all children with relevant symptoms, including females. Furthermore, we found that the clinical presentation of CASPR2 antibody-related neurological syndrome was highly heterogeneous and did not initially suggest the disease. It is important that the results presented in our case series and literature review indicate at least 1 of 7 symptoms can be identified in most patients before diagnosis: psychiatric abnormalities, sleep disorders, dysautonomia, movement disorders, seizures, neuropathic pain, and cognitive disturbances. These signs were often associated with other, less specific symptoms, such as muscle cramping or weakness, weight loss, headache, reduced consciousness, memory impairment, and fever. The clinical manifestations are consistent with those reported in prior studies [11–13]. So, we suggest the above points should increase suspicion of CASPR2 antibody-related neurological syndrome in children. The wide expression of CASPR2 in both the central and peripheral nervous system supports the various clinical conditions of this disease [14]. Interestingly, the most common presentation in CASPR2 antibody-related neurological syndrome in our cohort was psychiatric abnormalities, followed by sleep disorders and seizures, similar to prior pediatric literature [15]. As regards other symptoms, memory impairment, neuropathic pain and symptoms of PNH were less prevalent in our case series and literature review compared to adult series, possibly due to these symptoms being difficult to characterize in children [16, 17].

CASPR2 antibody-related neurological syndrome encompasses a wide clinical spectrum broadly divided into three overlapping autoimmune syndromes: autoimmune encephalitis, Morvan’s syndrome, and PNH. Our data support that AE (62.7%) is the most common phenotype of pediatric CASPR2 antibody-related neurological syndrome, followed by MoS (29.9%) and PNH (7.4%), consistent with the results in other observations [18–20]. Most of these syndromes show a substantial overlap of symptoms, although there are strikingly different relative frequencies of the overlapping features [13]. Sergio et al. found that symptoms of CASPR2 antibody-positive patients do not associate randomly, but they form preferential combinations instead [21]. In this case series and literature review, patients presenting mainly with AE showed more psychiatric abnormalities, seizures, and sleep disorders, but were less likely to develop dysautonomia, neuropathic pain, and weight loss. Some features that distinguished these patients from classical AE were the presence of neuropathic pain, weight loss, and male gender [22]. Moreover, CASPR2 antibody-related AE can present PNH, which is rarely seen in other encephalitis. These observations were in line with studies by Irani et al. and Qin et al. [14, 22]. Patients with MoS had symptoms of hyperexcitability of the peripheral nervous system, central nervous system, and autonomic system [23]. The main symptoms of MoS in our cohort are similar to those in the previous study, with patients exhibiting more cognitive disturbances, dysautonomia, and sleep disorders [23]. PNH is characterized by muscle twitching, cramps, and stiffness, along with paresthesia [14, 19]. Children with CASPR2 antibody-related PNH are under-recognized because symptoms of PNH are difficult to characterize, particularly in very young patients [24]. Electrodiagnostic studies play a major role in the initial diagnosis of PNH [25].

CASPR2 antibodies can be associated with tumors, but the incidence of association varies among different researchers [26]. Approximately 20% of adult patients with CASPR2 antibodies have an underlying neoplastic process, which in most cases can be identified as thymoma [27, 28]. In contrast, none of the 67 pediatric patients in our case series and literature review were diagnosed with neoplasm. This finding is consistent with that in previously reported pediatric cases [3, 29]. Therefore, the relationship between children with CASPR2 antibody-related neurological syndrome and neoplasm needs to be further confirmed with larger samples and longer follow-up time.

In contrast to adults, pediatric CASPR2 antibody-related neurological syndromes are rarely paraneoplastic. The underlying mechanism is more likely rooted in non-paraneoplastic immune activation. A post-infectious process, particularly via molecular mimicry, is considered the leading trigger [30]. Furthermore, the inherent instability of the developing immune system in childhood may predispose individuals to a break in self-tolerance following infections or other environmental triggers [31], facilitating the production of pathogenic antibodies in the absence of a tumor.

The CSF findings were unremarkable in the majority of patients. Unlike patients with anti-NMDAR or myelin oligodendrocyte glycoprotein antibody (MOG-Ab), who typically have inflammatory changes in the CSF (mildly raised cell count or protein levels) [32], only 37.5% of pediatric patients with CASPR2 antibody-related neurological syndrome in our case series and literature review have CSF abnormalities. Therefore, CSF may be most useful in helping to exclude other diseases. Considering CASPR2 antibodies, most studies suggest that serum testing has higher sensitivity than CSF [12, 33]. This was consistent with our patient for whom the serum CASPR2-IgG was positive but the CSF CASPR2-IgG was negative. Thus we need to pay more attention to the diagnostic value of serum antibodies. Irani et al. stated that about two-thirds of the patients have an abnormal EEG with diffuse or focal slowing [34], which is consistent with our findings. So, EEG changes involving a slow wave may be a diagnostic indicator of CASPR2 antibody-related neurological syndrome. Reviewing the literature, it is noted that less than one-third of patients with CASPR2 antibodies have MRI abnormalities [35], which can manifest in various regions of the brain [11], most frequently in medial temporal lobe flair high signal [36]. In our case series and literature review, brain MRI results were either normal or displayed only nonspecific abnormalities, with no definitive evidence indicating the most commonly affected region of the brain.

In terms of treatment and prognosis, there is no established treatment for CASPR2 antibody-related neurological syndrome. Immunotherapy is the primary treatment for all CASPR2 antibody-associated syndromes [37], leading to improvement in 93% of patients [38]. The most frequently used initial regimens were corticosteroids and IVIG [39], which were also seen in our case series and literature review. Second-line therapy was less commonly utilized, as the first-line treatment yielded a dramatic response in most cases [40]. Some cases of MoS can self-remit without immunotherapy [41], as illustrated by our case 2, though such rapid spontaneous remission as observed here has not been previously documented—the earliest reported remission occurring at 4 weeks [3]. Our case demonstrated recovery even earlier than 4 weeks without treatment, raising the question of a potential false positive antibody result. However, the anti-CASPR2 antibody was detected using a CBA, the current gold standard, at an accredited reference laboratory (Kindstar Global). The positive finding was further supported by characteristic clinical features, including cognitive impairment, peripheral nerve hyperexcitability, and dysautonomia, along with an EEG showing generalized slowing. Therefore, we consider this a true positive result, and well-documented cases like this are needed to establish the shortest possible time to spontaneous remission in untreated MoS. In addition, our case series and literature review found that 95.8% of pediatric patients with mRS ≤ 2 at last visit, which is higher than the previously reported range of 73–89% in adults [42, 43]. Furthermore, none of the patients in our cohort had a severe refractory disability at follow-up.

In summary, patients with CASPR2 antibody-related neurological syndrome are rare. By retrospective analysis of 67 pediatric patients, we recommended taking the CASPR2 antibodies test for all children with new-onset psychiatric abnormalities, sleep disorders, dysautonomia, movement disorders, seizures, neuropathic pain, and cognitive disturbances. Although this first case series and literature review highlights the great variability of clinical presentation associated with CASPR2 antibody-related neurological syndrome, for patients with different classifications of these conditions there is often a characteristic set of core phenotypic manifestations. Neoplastic processes are rare in pediatric patients. Early recognition of CASPR2 antibody-related neurological syndrome is important because it responds well to immunotherapy.

### Investigations

All patients exhibited positivity for anti-CASPR2 antibodies in CSF and/or serum. Specifically, 57 patients demonstrated the presence of antibodies in serum (with or without CSF), while 2 patients exclusively displayed antibodies in CSF. Additionally, 12 patients manifested positive CASPR2 antibodies in both their CSF and serum. Eight patients were exclusively assessed for the anti-CASPR2 antibodies, with no specific reference to its presence in the serum or CSF. Anti-CASPR2 antibody titers were available showing serum titers between 1:10 and 1:5120 and CSF titers between 1:2 and 1:16. No correlation between the titer and severity of symptoms was detected.

Of the 48 patients evaluated, CSF was normal in 30. Elevated CSF WBC counts, ranging from 6 to 84 cells/µL, were noted in 11 patients, and an increase in CSF protein (45–132 mg/dL) was detected in 10. Among the 18 cases with abnormal CSF findings, 13 (72.2%) were diagnosed with AE and 5 (27.8%) with MoS.

Among the 49 patients, EEG abnormalities were observed in 33 (67.3%), comprising 26 (78.8%) with AE and 7 (21.2%) with MoS. The most common finding was focal or generalized slowing (63.3%), while epileptiform discharges, predominantly sharp or spike waves, were present in 14.3% (*n* = 7) of the individuals.

Brain MRIs were abnormal in 14 of 58 patients (24.1%), including 11 cases (78.6%) of AE and 3 cases (24.1%) of MoS. The findings included: abnormal signals in various regions (brainstem, hippocampus, basal ganglia, temporal lobe, or parietal lobe; *n* = 1, each), white matter lesions (*n* = 4), atrophy (right hippocampal atrophy or brain atrophy; *n* = 1, each), meningeal enhancement (*n* = 2), and lacunar infarction (*n* = 1). Three additional cases had unspecified abnormalities.

Among the 67 cases, a subgroup of 32 underwent further screening with either tumor marker screening or radiological examination, and no tumors were detected. Furthermore, none of the 67 original case reports documented a tumor diagnosis.

### Immune therapy

Therapy data was available from 59 out of 67. All patients had clinical improvement during immunosuppressive treatment (*n* = 51) or symptomatic treatment (*n* = 8). The immunotherapy regimens for these 51 patients included one or more of the following: corticosteroid (*n* = 38), IVIG (*n* = 38), mycophenolate mofetil (MMF, *n* = 2), rituximab (RTX, *n* = 2), methotrexate (MTX, *n* = 1) and plasma exchange (PLEX, *n* = 2). Forty-six patients received first-line immunotherapy, whereas five received second-line immunotherapy. Among these patients, thirty-two of them received IVIG plus IVMP, six received IVIG alone, and six received IVMP alone. Symptomatic treatment alone was adopted in 13.6% of patients. The most frequent therapy was IVIG plus IVMP.

### Outcomes

After a median follow-up time of 13.0 months(rang 1–90.0), 57.1% of patients recovered completely, no patients died, and 5.0% of patients experienced a relapse. In our case series and literature review of 67 patients, 51 were treated with immunotherapy. Follow-up modified Rankin Scale (mRS) scores were available for 48 of these treated patients. Immunotherapy was associated with a favorable prognosis in most cases, with 46 patients (95.8%) achieving a favorable outcome (mRS score ≤ 2); the remaining 2 patients (4.2%) had an mRS score of 3. We did not observe any correlation between the age of onset and the outcome of the disease.

## Supplementary Information

Supplementary Material 1.

## Data Availability

The datasets used and/or analysed during this case series and literature review are available from the corresponding author on reasonable request.

## Abbreviations

AE: autoimmune encephalitis
CASPR2: Contactin-associated protein-like 2
CBA: Cell-based assay
CSF: Cerebrospinal fluid
CT: Computed tomography
EEG: Electroencephalogram
GCS: Glasgow Coma Scale
IVIG: Intravenous immunoglobulin
IVMP: Intravenous methylprednisolone
MMF: Mycophenolate mofetil
MOG-Ab: Myelin oligodendrocyte glycoprotein antibody
MRI: Magnetic resonance imaging
MRS: Modified Rankin score
MoS: Morvan syndrome
NCS: Nerve conduction study
PLEX: Plasma exchange
PNH: Peripheral nerve hyperexcitability
RTX: Rituximab
MTX: Methotrexate
