# Supplementary material for: CASPR2 antibody-related neurological syndromes in children: three cases report and literature review
Source: BMC Pediatr. 2026 Feb 5;26:216. doi: 10.1186/s12887-026-06549-4 (PMC12990615; doi:10.1186/s12887-026-06549-4)
Supplement: Supplementary file 1 — Supplementary Material 1. [file 12887_2026_6549_MOESM1_ESM.docx]

The cases presented in this article were derived from the following sources:

1. Tan C, Jiang Y, Zhong M, Hu Y, Hong S, Li X, Jiang L. Clinical Features and Outcomes in Pediatric Autoimmune Encephalitis Associated With CASPR2 Antibody. Front Pediatr. 2021 Oct 1;9:736035.
2. Kim SY, Choi SA, Ryu HW, Kim H, Lim BC, Hwang H, Chae JH, Choi J, Kim KJ, Hwang YS, Lee ST, Chu K, Lee SK. Screening Autoimmune Anti-neuronal Antibodies in Pediatric Patients with Suspected Autoimmune Encephalitis. J Epilepsy Res. 2014 Dec 31;4(2):55-61.
3. López-Chiriboga AS, Klein C, Zekeridou A, McKeon A, Dubey D, Flanagan EP, Lennon VA, Tillema JM, Wirrell EC, Patterson MC, Gadoth A, Aaen JG, Brenton JN, Bui JD, Moen A, Otten C, Piquet A, Pittock SJ. LGI1 and CASPR2 neurological autoimmunity in children. Ann Neurol. 2018 Sep;84(3):473-480.
4. Cheng YK, Ling YZ, Yang CF, Li YM. Contactin-associated protein-like 2 antibody-associated autoimmune encephalitis in children: case reports and systematic review of literature. Acta Neurol Belg. 2023 Oct;123(5):1663-1678.
5. Nagireddy RBR, Kumar A, Joshi D. Contactin-Associated Protein-Like 2 (CASPR2)-Associated Movement Disorder in a Child. Mov Disord Clin Pract. 2021 Aug 20;8(7):1153-1154.
6. Nosadini M, Toldo I, Tascini B, Bien CG, Parmeggiani L, De Gaspari P, Zuliani L, Sartori S. LGI1 and CASPR2 autoimmunity in children: Systematic literature review and report of a young girl with Morvan syndrome. J Neuroimmunol. 2019 Oct 15;335:577008.
7. Surana S, Kumar R, Pitt M, Hafner P, Mclellan A, Davidson J, Prabakhar P, Vincent A, Hacohen Y, Wright S. Acquired neuromyotonia in children with CASPR2 and LGI1 antibodies. Dev Med Child Neurol. 2019 Nov;61(11):1344-1347.
8. Nagarajan B, Gowda VK, Shivappa SK, Mahadevan A. CASPR2-Mediated Autoimmune Encephalitis in a Toddler. Indian Pediatr. 2020 Aug 15;57(8):757-758.
9. Wright S, Geerts AT, Jol-van der Zijde CM, Jacobson L, Lang B, Waters P, van Tol MJ, Stroink H, Neuteboom RF, Brouwer OF, Vincent A. Neuronal antibodies in pediatric epilepsy: Clinical features and long-term outcomes of a historical cohort not treated with immunotherapy. Epilepsia. 2016 May;57(5):823-31.
10. Suleiman J, Wright S, Gill D, Brilot F, Waters P, Peacock K, Procopis P, Nibber A, Vincent A, Dale RC, Lang B. Autoantibodies to neuronal antigens in children with new-onset seizures classified according to the revised ILAE organization of seizures and epilepsies. Epilepsia. 2013 Dec;54(12):2091-100.
11. Boesen MS, Born AP, Lydolph MC, Blaabjerg M, Børresen ML. Pediatric autoimmune encephalitis in Denmark during 2011-17: A nationwide multicenter population-based cohort study. Eur J Paediatr Neurol. 2019 Jul;23(4):639-652.
12. Miao A, Shi Y, Wang X, Ge J, Yu C. Clinical Features and Prognosis in Chinese Patients With Dipeptidyl-Peptidase-Like Protein 6 Antibody-Associated Encephalitis. Front Neurol. 2022 Jan 14;12:817896.
13. Kilic MA, Yoruk Yildirim ZN, Oner A, Yesil E, Aksu B, Yildiz EP, Yilmaz A, Caliskan MM. Pediatric LGI1 and CASPR2 autoimmunity associated with COVID 19: Morvan syndrome. J Neurol. 2021 Dec;268(12):4492-4494.
14. Nikolaus M, Jackowski-Dohrmann S, Prüss H, Schuelke M, Knierim E. Morvan syndrome associated with CASPR2 and LGI1 antibodies in a child. Neurology. 2018 Jan 23;90(4):183-185.
15. Rosenblatt T, Ort K, Shaw R, Levy RJ, Chen C, Niemi A, Hoang K. A Previously Healthy Adolescent With Acute Psychosis and Severe Hyperhidrosis. Pediatrics. 2020 Jun;145(6):e20193786.
16. Jiang Y, Tan C, Li T, Song X, Ma J, Yao Z, Hong S, Li X, Jiang L, Luo Y. Phenotypic Spectrum of CASPR2 and LGI1 Antibodies Associated Neurological Disorders in Children. Front Pediatr. 2022 Apr 7;10:815976.
17. Kang Q, Liao H, Yang L, Fang H, Hu W, Wu L. Clinical Characteristics and Short-Term Prognosis of Children With Antibody-Mediated Autoimmune Encephalitis: A Single-Center Cohort Study. Front Pediatr. 2022 Jul 8;10:880693.
18. Zhang J, Ji T, Chen Q, Jiang Y, Cheng H, Zheng P, Ma W, Lei T, Zhang Y, Jin Y, Wei C, Wu Y, Chang X, Bao X, Zhang Y, Xiong H, Ji X, Feng S, Ren H, Yang J, Jiang Y. Pediatric Autoimmune Encephalitis: Case Series From Two Chinese Tertiary Pediatric Neurology Centers. Front Neurol. 2019 Aug 22;10:906.
19. Wang Jue, Xin Lin, Lin Xinfu, Mei Ainong. Anti-Caspr2 antibody-associated encephalitis in a child: a case report and literature review. J Clin Pediatr. 2019 Jan 37(1):47-50.
20. Qiao S, Zhang SC, Zhang RR, Wang L, Wang ZH, Jiang J, Wang AH, Liu XW. Thyroid Function and Low Free Triiodothyronine in Chinese Patients With Autoimmune Encephalitis. Front Immunol. 2022 Feb 10;13:821746.
21. Rosch RE, Bamford A, Hacohen Y, Wraige E, Vincent A, Mewasingh L, Lim M. Guillain-Barré syndrome associated with CASPR2 antibodies: two paediatric cases. J Peripher Nerv Syst. 2014 Sep;19(3):246-9.
22. Syrbe S, Stettner GM, Bally J, Borggraefe I, Bien CI, Ferfoglia RI, Huppke P, Kern J, Polster T, Probst-Müller E, Schmid S, Steinfeld R, Strozzi S, Weichselbaum A, Weitz M, Ziegler A, Wandinger KP, Leypoldt F, Bien CG. CASPR2 autoimmunity in children expanding to mild encephalopathy with hypertension. Neurology. 2020 Jun 2;94(22):e2290-e2301.
23. Koshy KG, Iype T, Panicker P. Stimulus-Induced Motor Afterdischarges in CASPR2 (Contactin-Associated Protein-Like 2)-Positive Peripheral Nerve Hyperexcitability Syndrome. Cureus. 2023 Sep 20;15(9):e45643.
24. AlHakeem AS, Mekki MS, AlShahwan SM, Tabarki BM. Acute psychosis in children: do not miss immune-mediated causes. Neurosciences (Riyadh). 2016 Jul;21(3):252-5.
